# Arsenic in Water and Food: Toxicity and Human Exposure

**DOI:** 10.3390/foods14132229

**Published:** 2025-06-24

**Authors:** Pierina Visciano

**Affiliations:** Department of Bioscience and Technology for Food, Agriculture and Environment, University of Teramo, Via R. Balzarini 1, 64100 Teramo, Italy; pvisciano@unite.it

**Keywords:** drinking water, rice, seafood, speciation, methylation, estimated daily intake, margin of exposure

## Abstract

Arsenic is a human carcinogen present in drinking water and food, especially rice, rice products and seafood. It can be found in both organic and inorganic forms, the latter being the most toxic. In addition to the carcinogenic effect, exposure to inorganic arsenic can cause numerous disorders in different organs/systems of the human body, such as the skin, cardiovascular, neurological, endocrine, immune, and reproductive systems. The risk assessment associated with dietary arsenic is mainly based on the margin of exposure, i.e., the ratio between the dose at which a small but measurable adverse effect may occur and the estimated daily intake of the target substance. It is mainly influenced by arsenic concentrations and consumption data of average or 95th percentile consumers. This review focuses on the toxicity of arsenic, its sources and routes of human exposure, with particular attention to the ingestion of contaminated water and food, considering the differences between age groups and dietary habits.

## 1. Introduction

Arsenic (As) is released into the environment from both natural sources (e.g., degradation and dissolution of rocks/minerals, and volcanic emissions) and anthropogenic activities, such as mining, coal and geothermal exploitation, agricultural practices, industrial and municipal waste, fertilizers, pesticides, paints, and cosmetics [1]. It can contaminate groundwater and surface water, as well as soil and air, resulting in human exposure mainly through the intake of contaminated drinking water and food [2]. It ranks first in the list of substances included in the National Priority List (NPL) published by the Agency for Toxic Substances and Disease Registry (ATSDR). The substance prioritization algorithm is based on the frequency of occurrence, toxicity and potential for human exposure [3]. In the environment (e.g., soil and groundwater), it is mainly found as inorganic As (iAs) divided into arsenite As(III) and arsenate As(V), while in terrestrial and marine organisms, as well as in algae, organic As species are prevalent [4].

There are two groups of organic As compounds, the small methylated species that usually contain oxygen or sulfur in their structure and derive from methylation through the substitution of a hydroxyl ligand with a methyl group (Table 1), while by replacing one or more methyl or hydroxyl ligands with sugars, lipids or cyclic groups, many other organic As compounds are obtained, such as arsenobetaine (AsB), arsenocholine (AsC), arsenosugars (AsSs), and arsenolipids (AsLs) [5]. It is necessary to specify that the acronyms MMA and DMA are used to indicate, respectively, all monomethylated and dimethylated As species (including thio-arsenical species) [6]. For complex organic species, both AsB and AsC contain an alkyl chain with 2–4 carbon atoms, while AsSs contain a ribose sugar and AsLs contain > 10 carbon atoms [7].

As(V) forms are more stable than As(III), which on the contrary are labile in solution and easily convert into As(V), thus generating problems in food analysis [6]. Due to the different bioavailability and toxicity of As forms, the development of analytical techniques not only for the determination of total As concentrations, but also for the selective separation of all As species, is particularly significant. The speciation analysis is able to differentiate the numerous chemical species of an element in the target matrix [4]. The most common method for As speciation is high performance liquid chromatography (HPLC) with inductively coupled plasma mass spectrometry (ICP-MS), although it requires the use of reference standards for analyte identification. Recently, electrospray ionization mass spectrometry (ESI-MS) combined with ICP-MS can identify and quantify even new unknown As species by their fragmentation and molecular ion information. However, since different As forms exhibit different ionization efficiencies, its application is not able to identify all As species and needs to be complemented with efficient separation techniques, depending on their physicochemical properties. In particular, positively and negatively charged As species are well separated by cation and anion exchange chromatography, respectively. A new method based on hydrophilic interaction liquid chromatography (HILIC) can simultaneously separate neutral, cationic, and anionic As species and is therefore particularly advantageous in As speciation where all these types of species may be present [8].

**Figure 1:**
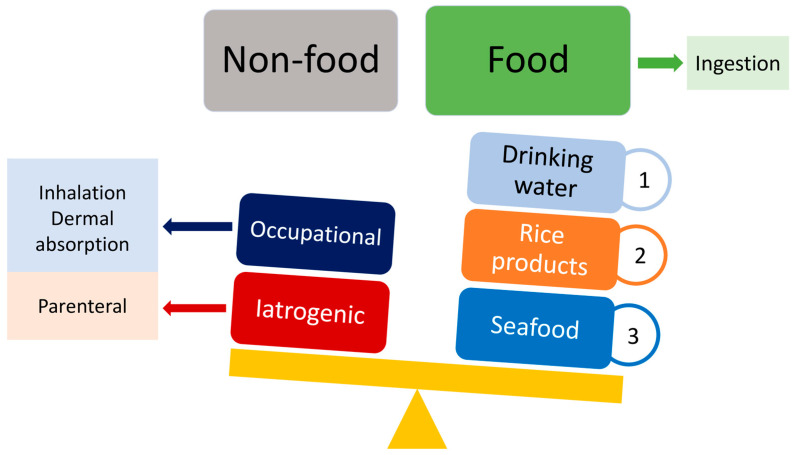
Sources of arsenic and human exposure. Ingestion of contaminated food is prevalent compared to other routes.

Human exposure to As can occur through ingestion of contaminated drinking water and food and/or inhalation by individuals working or living in proximity to certain industries such as wood preservatives, pigments, pharmaceuticals, and pesticides, as well as smelting and mining activities [5]. Additional arsenicals such as arsenic trioxide and S-dimethylarsino-glutathione are used in the treatment of acute promyelocytic leukemia and refractory solid tumors, respectively [9]. The ranking order of sources and routes of human exposure to As is shown in Figure 1. The ingestion of contaminated water and food is the main contributing factor, while occupational and iatrogenic exposure is limited to single individuals. People living in areas with high As concentrations in groundwater and, consequently, in soil and growing plants, are most at risk through dietary intake.

**Figure 2:**
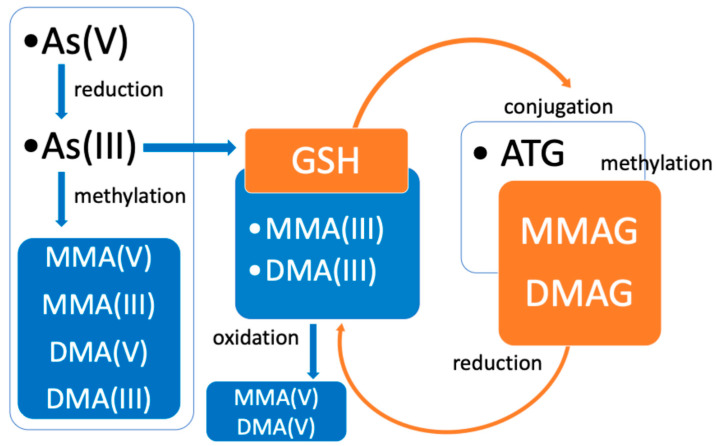
Metabolism of inorganic arsenic in humans: As(V), arsenate; As(III), arsenite; MMA(V), monomethylarsonic acid; MMA(III), monomethylarsonous acid; DMA(V), dimethylarsinic acid; DMA(III), dimethylarsinous acid; GSH, glutathione; ATG, arsenic triglutathione; MMAG, monomethylarsonic diglutathione; DMAG, dimethylarsinic glutathione [12].

After ingestion, approximately 70–90% of iAs(III) and iAs(V) is absorbed from the gastrointestinal tract and distributed to different tissues depending on the protein type. Trivalent arsenicals bind to sulfhydryl groups of keratin proteins resulting in high As concentrations in hair, skin, and nails [10]. Metabolism of iAs occurs primarily in the liver through two major pathways: reduction/oxidation reactions that interconvert iAs(III) and iAs(V), and methylation reactions that convert As(III) to the methylated species [11]. Another proposed pathway suggests that iAs is conjugated with glutathione and subsequently methylated by S-adenosylmethionine methyltransferase. More specifically, As triglutathione (ATG) is methylated to monomethylarsonic diglutathione (MMAG) and dimethylarsinic glutathione (DMAG), which are further reduced to MMA(III) and DMA(III) and then oxidized to their respective pentavalent forms (Figure 2) [12]. However, there is a large individual variability in As methylation, probably due to a large difference in methyltransferase activity and possible polymorphism [13]. Excretion of As compounds occurs primarily in the urine as iAs (10–30%), monomethylated As forms (10–20%), and dimethylated As forms (60–80%) [14].

Several reviews on the occurrence of As in the environment and the food chain have been published, addressing human As intake and its adverse health effects. The main objective of this review was to evaluate the toxicity of As and human exposure in the light of recent risk assessment studies, based not only on concentrations of undesirable/toxic substances, but also taking into account additional significant factors influencing the total intake and risk characterization, such as dietary habits (as average or heavy consumers), the influence of body weight (bw) and threshold limits established by scientific expert communities and international institutions.

## 2. Toxicity and Regulatory Limits

The toxicity of As varies with its chemical structure and oxidation state. Inorganic As is more toxic than organic forms, and trivalent species are more toxic than pentavalent ones [5]. The classification of the International Agency for Research on Cancer (IARC) includes iAs compounds, in particular As(III), As(V) and arsenic trioxide in Group 1 of substances with sufficient evidence of human carcinogenicity, MMA and DMA in Group 2B (possibly carcinogenic to humans), while AsB and organic As compounds are in Group 3, which is not classifiable [15]. Many epidemiological studies have demonstrated the association between human exposure to iAs and the development of tumors in some districts (e.g., lung, bladder, and skin), but not in others (breast, prostate, liver, and pancreas) [12]. This is more evident in some countries of Asia and South America, where drinking water is particularly contaminated by high concentrations of iAs (≥150 μg/L). A comprehensive review that considered 35 years of global epidemiological studies suggested that exposure to 10, 20, 50 and 150 μg/L of As was associated with an 11, 32, 67 and 121% increased risk of lung cancer, respectively. Furthermore, individuals exposed to 20 μg/L may have a 22% increase in lung cancer mortality [16]. A large population-based study has shown that levels at or below 20 μg/L cause an increase in lung cancer mortality [17]. Begum et al. (2015) reported 4.51 and 2.91 additional cases of lung and bladder cancer per 100,000 people exposed to 10 μg/L in drinking water, respectively [18]. Koutros et al. (2018) investigated the relationship between cumulative As intake from drinking water and risk of bladder cancer in a population-based case–control study in New England [19].

It has been reported that iAs does not interact directly with DNA, but its methylated forms can induce oxidative stress. The cellular metabolism of iAs includes methylation in the four abovementioned species, namely MMA(V), MMA(III), DMA(V), and DMA(III). The most cytotoxic is MMA(III), which can inhibit mitochondrial processes and lead to the formation of reactive oxygen or nitrogen species [20]. This production of free radicals can cause oxidation of DNA bases and also DNA single- and double-strand breaks [21]. The genotoxic potential of iAs has been demonstrated by the inhibition of DNA repair both in vitro and in vivo [12]. Genome-unstable conditions caused by impaired DNA repair may affect the normal expression of cancer-related genes [13]. One of the most recently studied mechanisms by which iAs can cause cancer is the dysregulation of microRNAs (miRNAs), i.e., small non-coding RNAs involved in both normal and pathological functions, such as cell cycle regulation, differentiation, proliferation, apoptosis, stress tolerance, as well as carcinogenic potential as oncogenes or tumor suppressor genes. Some studies have shown that As can induce changes in miRNA expression [22].

Chronic exposure to iAs induces a wide range of clinical pathologies such as diabetes mellitus, liver and kidney dysfunction, cardiovascular outcomes, peripheral neuropathy, cognitive impairment, and reproductive complications. In addition, it can accumulate in the skin, causing arsenical keratosis and hyperpigmentation [23]. Most of the toxic effects caused by iAs in humans are reported in Table 2 [24].

A strong association between iAs exposure and cardiovascular diseases has been observed, particularly for myocardial infarction in males and stroke in females [25]. Adverse effects on the central nervous system may result in physical growth retardation, cognitive dysfunction and neurodegenerative diseases [26]. Disturbances of the oxidative/antioxidant balance and memory impairment have also been reported [27]. Regarding the immune system, iAs may alter its homeostasis through two potential effects, namely loss of response (immunosuppression) resulting in high susceptibility to infections or exacerbation of the response (immunostimulatory autoimmunity) [28].

**Figure 3:**
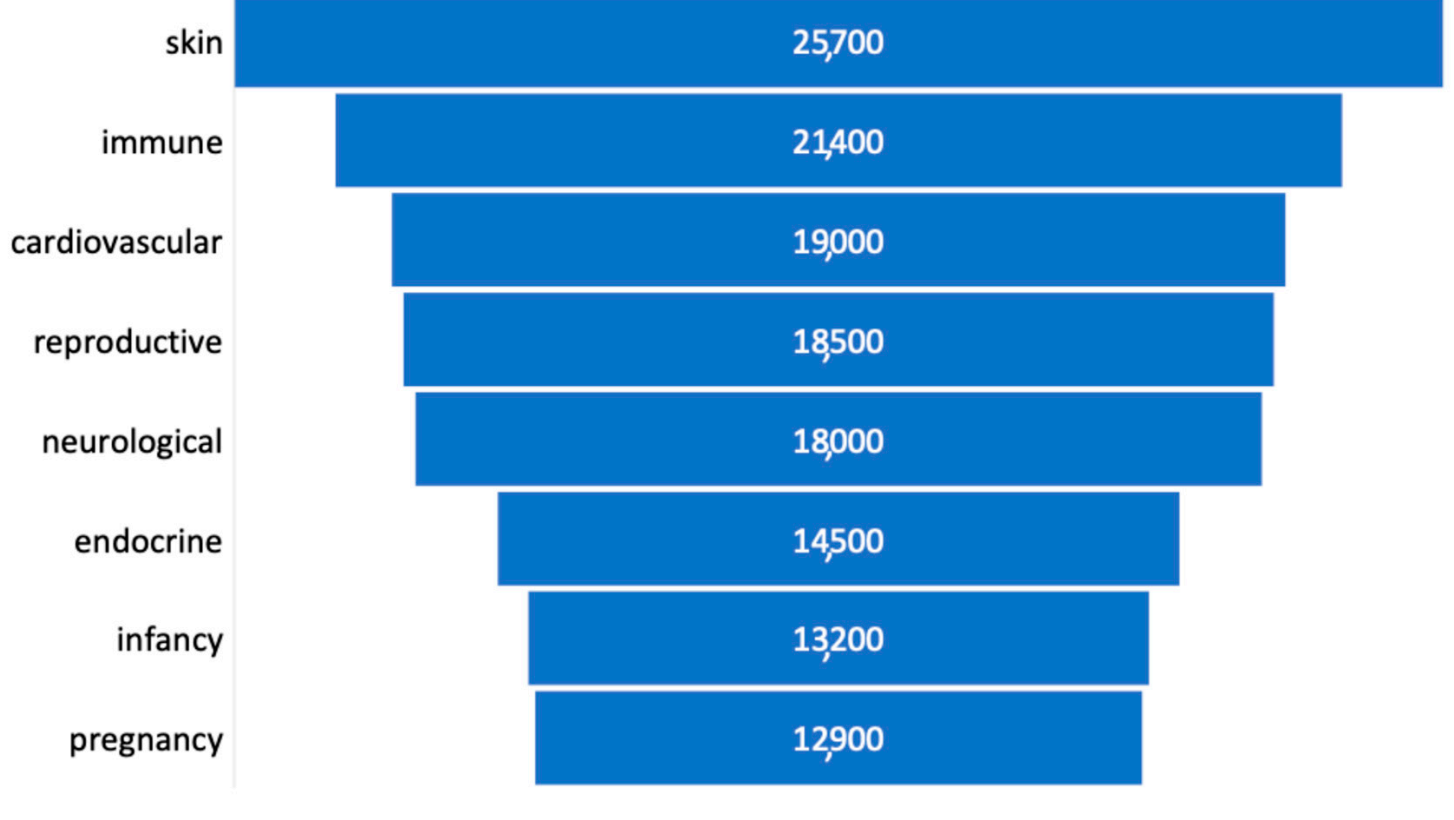
Number of research articles reported by Google Scholar in the last thirty years concerning the arsenic toxic effects in human body systems or in a specific life stage.

Studies on human exposure to iAs during pregnancy have shown that it can easily cross the placenta, causing abortion, fetal death, premature birth, or low birth weight [29]. During infancy, iAs may induce skin alterations, immune suppression, cognitive impairment, and enzyme inhibition [30]. In Figure 3, the number of research articles reported by Google Scholar on iAs toxicity in various human body systems or in a particular life stage, such as infancy and pregnancy, is shown in decreasing order.

Regarding carcinogenicity, Google Scholar reports approximately 52,000 scientific publications over the last thirty years. Based on its carcinogenic potential, the reference value in drinking water has been reduced from 50 to 10 µg/L, following the possibility of detecting at low concentrations thanks to the progress of analytical techniques [31]. The maximum limit of 10 µg/L is also established in the United States [32] and the European Union (EU) for potable water [33] as well as in most countries worldwide. Only a few States on five continents still apply the upper limit of 50 µg/L. Interestingly, the 10 and 50 μg/L limits are estimated to lead to 2500 and 12,000 excess cancer deaths per 1,000,000 people, respectively [34].

In EU Member States, maximum levels for iAs are also reported in some food categories (Table 3). Foods exceeding these limits cannot be placed on the market nor can they be used as ingredients in processed products [35].

Paddy rice (rice grain) is rice (*Oryza sativa* L.) that has retained the husk after threshing, while husked rice (brown rice) is paddy rice from which only the husk has been removed. During husking and handling of rice some loss of bran may occur. The term polished rice (white rice) refers to husked rice from which all or part of the bran and germ have been removed by milling [36]. The maximum limits for infant formulae, follow-on formulae, and baby food (0.020 and 0.010 mg/kg) are much lower than those of other foods, since the dietary exposure to iAs for children under three years of age is generally estimated to be about 2 to 3-times higher than that of adults on a body weight (bw) basis. Furthermore, rice is an important ingredient in a wide range of food products such as rice waffles, rice wafers, rice crackers and rice cakes. The lower limit of 0.020 mg/kg has also been established for fruit juices and fruit nectars, since they are mainly consumed by this age group [35]. 

According to the Codex Alimentarius Commission, the maximum limits for iAs are 0.20 and 0.35 mg/kg for polished rice and husked rice, respectively. Countries may decide to use their own screening procedure by analyzing the total As content and, if its concentration is lower than or equal to the maximum reference limit, no further analysis is required, while if this value is higher, follow-up analyses should be performed [37]. In the United States, the Food and Drug Administration (FDA) can set an action level for a hazardous substance, indicating the degree of contamination at which a food may be considered a health concern. Action levels are used as guidelines for the food industry, rather than as tolerance or regulatory limits, and can be achieved by applying good manufacturing practices, particularly by selecting and testing rice or rice-derived ingredients with low concentrations of iAs. The action levels for iAs in infant rice cereals and apple juice are 100 and 10 μg/kg, respectively [38,39].

## 3. Literature Studies on Arsenic Presence in Water and Food Samples

**Figure 4:**
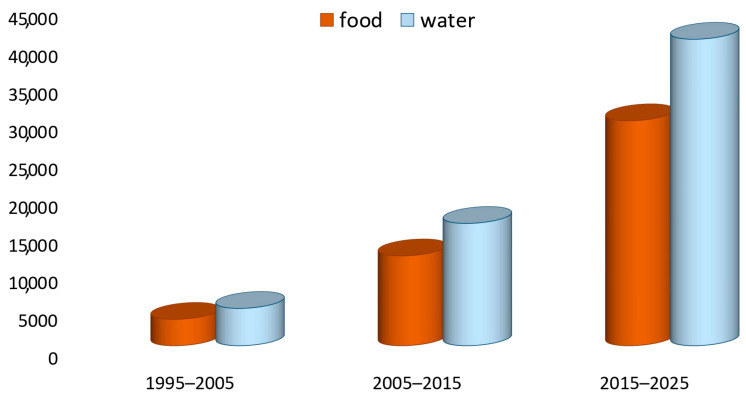
Number of research articles reported by Google Scholar in the last three decades obtained by associating the terms “arsenic and food” and “arsenic and water”.

A literature search by Google Scholar on scientific publications regarding the presence of As in water and food samples in the last thirty years (from 1995 to 2025) highlighted an increasing trend with a greater number of articles related to water than to food (Figure 4). 

**Figure 5:**
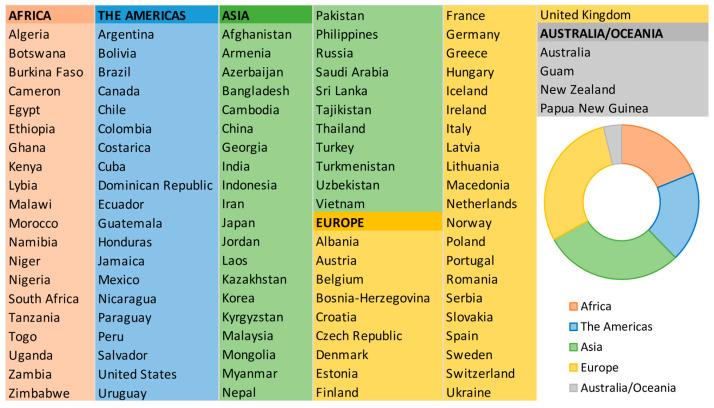
Countries in five continents with inorganic arsenic content above 10 μg/L in groundwater [41].

Drinking water has been reported as the major source of human dietary exposure, with iAs being the most prevalent, while both inorganic and organic forms may be present in foods [40]. In natural water, its concentrations are generally 1–2 μg/L; although, in groundwater where there are mineral deposits deriving from volcanic rocks, they can be significantly higher [14]. The natural occurrence of iAs levels above 10 μg/L in groundwater due to geological characteristics of several countries around the world is shown in Figure 5 [41].

Several studies have confirmed the presence of high iAs concentrations in drinking water. A total of 356 (4.7%) samples out of 7623 from different EU Member States showed iAs levels exceeding the maximum limit of 10 µg/L [42]. It should be noted that drinks, soups and similar dishes prepared with drinking water may also represent an additional source of dietary iAs intake [14].

Where contaminated water is widely used for irrigation, soil becomes the main route of iAs contamination in crops [43]. All As species can be transferred to plants through specific transporter proteins. However, the uptake amounts of iAs species are higher than those of MMA, DMA, and other organic forms [44]. Rice has a high capacity to accumulate iAs in both shoot and grain, with a concentration 10 times higher than other crops [45]. Many studies have reported that rice is more contaminated due to anaerobic and flooded growth conditions [46], as well as highly expressed plant cell-specific carrier proteins [47]. The mean transfer factor is about 0.8 in rice, while it corresponds to 0.2 and 0.1 in barley and wheat, respectively. Since rice is grown with different periods of submerged and dry conditions, an alternation of oxidative and reductive processes can occur in the soil. Therefore, in aerobic and anaerobic environments, iAs is present mainly in the oxidized or reduced forms as As(V) or As(III), respectively [48].

Post harvest treatments, such as husking, polishing, and parboiling, can influence the iAs content in rice grains [49]. Polished (white) rice contains less iAs than husked (brown) rice due to the polishing treatment which removes the bran layer containing most of the iAs. It has been shown that iAs can be ten times higher in unpolished than polished rice [50]. Atiaga-Franco et al. (2019) [51] found mean total As concentrations of 0.232 and 0.174 mg/kg in brown and white rice samples from Ecuador, respectively. The higher As concentration in brown rice was probably due to the presence of the outer bran layer of the rice grain. Li et al. (2015) [52] also reported higher mean total As levels in unpolished rice grains compared to polished ones from different regions (0.302 and 0.151 mg/kg in Guangxi rice, or 0.200 and 0.085 mg/kg in Yunnan rice, respectively). They found that the reduction in total As during the polishing process was greater for iAs than for the organic forms. Similarly, González et al. (2020) [53] detected maximum total As and iAs levels (0.229 and 0.190 mg/kg, respectively) in brown rice products compared to white rice samples (0.151 and 0.090 mg/kg, respectively). In another study, infant foods containing rice were found to be contaminated with mean total and iAs concentrations of 0.205 and 0.152 mg/kg in brown rice, and 0.143 and 0.094 mg/kg in white rice, respectively [54].

The iAs levels in cooked rice are largely dependent on the quality of the water used for cooking, some pre-cooking practices (washing and soaking), and cooking methods. Cooking rice with large amounts of water and then discarding the excess water may reduce the iAs content, although its potential presence in the water may influence this outcome [36]. Halder et al. (2014) [55] showed that cooking rice with iAs-safe water resulted in an average reduction of 42.1% compared to raw rice. In contrast, iAs content increased by an average of 78.5% due to cooking with contaminated water. In another study, pre-rinsing raw rice and boiling with excess water was found to be the most efficient means of achieving significant iAs removal, particularly in white rice varieties [56].

Total As is also found in seafood, including fish and shellfish, in concentrations up to 100 times higher than in other types of contaminated foods such as rice. It is absorbed mainly by phytoplankton and/or bacteria and rapidly metabolized into organic forms, which are the dominant species in marine organisms [57]. More specifically, As(V) is reduced to As(III), which is quicky detoxified through methylation in methylated organic species and AsSs. The enzyme S-adenosylmethionine acts as a methyl and ribosyl (sugar) groups donor. AsB is the end product synthesized from AsC, which serves as a precursor. The analysis of 80 samples, including molluscs, echinoderms, and crustaceans, showed presence of AsB at concentrations ranging from 0.744 mg/kg in octopus to 5.28 and 12.4 mg/kg in shrimp and crab, respectively. The content of iAs was calculated as the sum of As(III) and As(V) corresponding to 0.203 and 0.044 mg/kg in marine and freshwater river giant prawns, respectively [58]. Other authors reported mean total As concentrations of 2.82 and 1.71 mg/kg in marine fish and seafood, respectively. The contents of AsB, MMA and DMA were 2.39, 0.0400 and 0.229 mg/kg in marine fish and 1.22, 0.226 and 0.161 mg/kg in seafood, respectively [59]. AsSs are the predominant arsenicals present in macroalgae, but they have also been reported in molluscs, gastropods, and crustaceans. AsLs represent up to 70% of the total As content in seafood [60].

## 4. Human Exposure and Risk Assessment

Dietary exposure to chemical contaminants depends on their levels in food and the dietary habits of average or high consumers (95th percentile) [61]. The estimated daily intake (EDI) is obtained by calculating it according to the following formula:

$$EDI=\frac{concentrations\times foodconsumption\left(\frac{g}{day}\right)}{bodyweight}$$

where

EDI = estimated daily intake

Several studies have been reported in the literature regarding dietary As intake, showing that it is influenced not only by contamination levels but also by daily food consumption, which varies significantly from country to country across the world [62,63]. The dietary habits of Asian populations, who consume rice as a primary crop, can significantly influence EDI. Islam et al. (2017) [64] studied iAs occurrence in rice from different districts of Bangladesh and found that EDI varied from 0.38 to 1.92 μg/kg bw, while another study conducted on Chinese population reported EDI values of 0.79 and 0.41 μg/kg b.w. for unpolished and polished rice consumption, respectively [52]. Exposure to iAs calculated in adults using consumption data from 19 European countries ranged from 0.13 to 0.56 μg/kg bw and from 0.37 to 1.22 μg/kg bw per day for average and 95th percentile consumers, considering the lower bound (LB) and upper bound (UB) approaches, respectively. Children under three years of age were the most exposed, with dietary intakes ranging from 0.50 to 2.66 μg/kg bw [65]. It should be noted that in the LB approach, results below the limit of detection (LOD) are blanked and results below the limit of quantification (LOQ) are replaced by the value reported as LOD, while in the UB, results below the LOD are replaced by the value reported as LOD and results below the LOQ by the value reported as LOQ [66].

Certain groups of people are particularly vulnerable to As exposure, including pregnant women, developing children, and those with underlying genetic risk factors. In addition to the direct influence of bw on EDI, children under three years of age may be more exposed to As than adults due to high consumption of infant rice products. Other groups, such as people with celiac disease or gluten intolerance/sensitivity, may be more exposed due to high rice consumption [12]. Several studies confirmed a higher EDI for As in infants and children compared to adults. Cubadda et al. (2016) [67] reported EDI values of 0.190 and 0.152 μg/kg bw in Italian infants (1–3 years) and children (3–10 years), respectively, while it corresponded to 0.070 μg/kg bw in adults. Dietary iAs exposures calculated from total As concentrations by applying a 70% conversion factor were 0.20 and 0.08 μg/kg bw in infants (1–2 years) and children (6–11 years), respectively, and 0.06 μg/kg bw in adults (35–50 years) belonging to the German population [66]. Boon et al. (2022) [68] reported EDI values of 0.10 and 0.31 μg/kg bw (LB and UB, respectively) in infants (1–2 years) living in the Netherlands. Food subgroups contributing more than 15% of iAs intake were rice and concentrated fruit juices. A survey of chronic dietary exposure to iAs in the European population showed that the highest exposure levels were observed in infants and toddlers, corresponding to 0.22–0.61 and 0.30–0.61 μg/kg bw (LB and UB), respectively. At the 95th percentile, the highest EDI values were 0.52–1.20 and 0.58–0.99 μg/kg bw (LB and UB) in infants and toddlers, respectively. Indeed, the highest reported exposure in adults ranged from 0.07 to 0.15 (LB and UB) as average consumers, and between 0.19 and 0.33 μg/kg bw (LB and UB) at the 95th percentile. The most representative foods were rice and rice products, other cereals and derived products, and drinking water. Cereal-based foods for infants and young children, as well as biscuits, rusks and shortbread for children, also showed a significant dietary contribution [42].

Toxicity studies aim to identify a reference point (RP) to assess the level of human intake of a hazardous substance at which no appreciable adverse health effects are confidently predicted to occur. The application of the benchmark dose (BMD) is based on the amount that causes a low but measurable effect on a target organ and can be considered as an alternative to the traditional no observed adverse effect level (NOAEL) approach. For genotoxic and carcinogenic substances, the NOAEL cannot be used as an RP and the BMD is the preferred methodology to calculate the margin of exposure (MOE) for such compounds [69]. In 2009, the European Food Safety Authority (EFSA) Panel on Contaminants in the Food Chain (CONTAM Panel) published a report on As in food, stating that the previous provisional tolerable weekly intake of 15 μg/kg bw could no longer be considered appropriate due to the carcinogenic potential of iAs. The CONTAM Panel selected a lower confidence limit for the benchmark dose (BMDL_01_) of 0.3 to 8 μg/kg bw per day for a 1% increase in risk of lung, skin and bladder cancer, as well as skin lesions [65]. In the recent update of the iAs risk assessment published by EFSA in 2024 [12], the CONTAM Panel assessed a large number of scientific studies on the toxic effects of iAs and established a BMDL_05_ of 0.06 μg iAs/kg bw per day, based on a 5% benchmark response from a case–control study of skin cancer. Since iAs is considered a genotoxic carcinogen and therefore a health-based guidance value cannot be established, it is more appropriate to apply the MOE approach for risk characterization. The MOE can be calculated according to the following formula:

MOE = BMDL_05_/EDI

where

MOE = margin of exposure
BMDL = benchmark dose lower confidence limit
EDI = estimated daily intake

**Figure 6:**
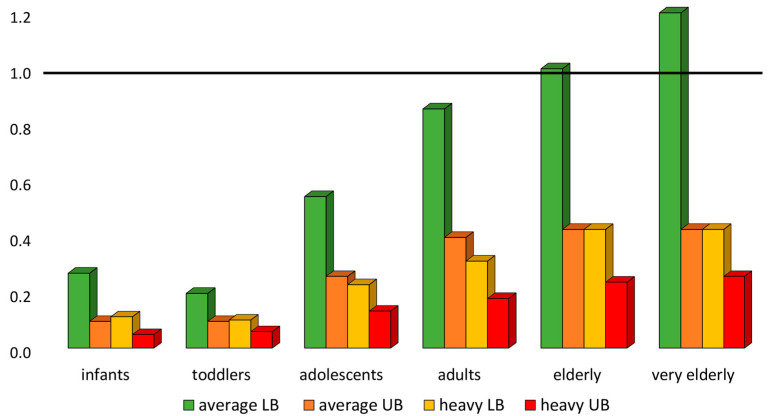
Margin of exposure for inorganic arsenic calculated in different age groups distinguished as average and heavy consumers (95th percentile) considering both the lower bound (LB) and upper bound (UB) approaches [39]. The black line represents the value of margin of exposure below which a high risk concern is considered.

It provides an estimate of how much lower the expected exposure to iAs is than the BMDL_05_ and, consequently, a low MOE represents a greater risk than a high MOE [61]. Considering dietary exposure estimates for average adult consumers (0.03–0.15 μg/kg bw) and at the 95th percentile (0.07–0.33 μg/kg bw) in the abovementioned risk assessment study [42], the MOE values ranged between 2 and 0.4 and between 0.9 and 0.2, respectively. Since MOE of 1 is the level of exposure that could be associated with a 5% increase from the background incidence of skin cancer, the CONTAM Panel concluded that some findings raised health concerns [12]. In Figure 6, MOE values calculated by dividing the BMDL_05_ (0.06 μg/kg) by the highest EDI (LB and UB) for average and heavy consumers of all age groups, obtained from 44 different dietary surveys carried out in 23 different European countries [42], are shown. The age groups included infants (1–3 years), toddlers (4–9 years), adolescents (10–17 years), adults (18–65 years), elderly (>65 years) and the very elderly (>75 years). The MOE results were always lower than a value of 1, which is considered a low health risk for iAs exposure, except for average consumers (LB) of the elderly and very elderly age groups. Therefore, all results < MOE could be assessed as a health risk. These data demonstrated a higher risk of adverse health effects particularly in infants and young children, who showed the lowest MOE, because rice and rice products represent a greater potential source of dietary exposure for these age groups than for adults and also because they consume more food relative to their bw compared to adults. In addition, infants and children may be particularly susceptible to the adverse neurodevelopmental effects [38]. The results of quantitative risk assessment studies should provide a means to plan interventions and mitigation measures to prevent and reduce dietary exposure to iAs. In such cases, specific advice on dietary recommendations to consumers could be considered to complement regulatory measures when these are not sufficiently adequate to protect public health and food safety. National or competent food control authorities should consider sharing information on the risks and benefits of consuming polished and/or husked rice in relation to As contamination, implementing practices that reduce its concentration during processing and cooking, such as cooking rice in large quantities of water and then discarding the excess water. For the most at-risk groups, such as infants and children, the importance of varying the diet by using, for example, cereals other than rice, may represent a significant difference in dietary intake of iAs [36].

Furthermore, efforts should be made to reduce uncertainties in the assessment of dietary exposure to iAs by performing speciation analyses and collecting food consumption data for both average and heavy consumers, with particular attention to the age groups at highest risk, such as infants, children, and pregnant women. However, some uncertainties may arise from model selection on risk estimates, in addition to those deriving from food contamination levels, sampling and consumption data or dose–response relationships. The best estimate of dietary intake is based on the national dietary pattern and corrections for variations in iAs concentrations during food preparation, which may be due to cooking methods, potential iAs presence in water used for cooking and other differences in household practices [70].

EFSA has recently also studied organic forms of As in relation to human dietary exposure. A risk assessment study conducted by the CONTAM Panel on methylated As species in food proposed a BMDL_10_ of 18.2 and 1.1 mg/kg bw for MMA(V) and DMA(V), corresponding to 9.7 and 0.6 mg As/kg bw per day, respectively. These criteria were considered as RP for the two small organo-arsenic species, while for the other organic forms, the toxicological studies were not sufficient to identify their toxic effects. The results of analysis for DMA(V) and MMA(V) content in food samples collected from eight and four EU countries, respectively, showed the highest estimated chronic dietary exposure to DMA(V) in toddlers (0.130–0.157 and 0.397–0.477 μg As/kg bw, LB-UB and average and 95th percentile, respectively), while in adults they corresponded to 0.038–0.044 and 0.133–0.158 μg As/kg bw for average and 95th percentile, respectively. The main risk factors were fish and seafood, cereals and cereal products for all age groups, as well as food products for young populations, such as infants and toddlers. For MMA(V), the highest EDI (0.342 μg As/kg bw) was obtained for heavy consumers of fish and processed fish, in infants and elderly age groups, respectively. Consequently, the MOE approach for risk characterization was applied, considering values of 500 and 10,000 for MMA(V) and DMA(V), respectively. The MOE calculation for MMA(V) exceeded 500 for both average and heavy consumers and therefore did not raise health concerns. Indeed, MOE values for DMA(V) were below 10,000 for some exposures at the 95th percentile, representing health concerns, although genotoxicity and carcinogenicity associated with DMA(V) exposure are not fully understood [6].

The EFSA CONTAM Panel has assessed a risk characterization also for the complex organo-arsenic species, such as AsB, AsSs and AsLs. Since AsB showed no adverse effects in animal and human studies, there is sufficient evidence that it is not of health concern. AsSs are the major As species in marine seaweeds, particularly in brown seaweeds. For glycerol arsenosugar, the BMDL_10_ of 0.85 mg As/kg bw per day resulted from the impairment of cognitive and motor functions observed in mice. The highest EDI calculated for adult consumers of red seaweed at the 95th percentile was 0.71 μg As/kg bw, which corresponded to MOE > 1000 considered not to be of health concern. Finally, no risk characterization could be performed for AsLs due to lack of data [7].

## 5. Conclusions

Human exposure to As is a global health concern, as people use groundwater for drinking, cooking, and other domestic purposes, as well as for agricultural activities such as irrigation and flooding. Major dietary risk factors include cereals and cereal products, especially rice due to its higher iAs content compared to other crops, and fish and seafood, which are particularly rich in organic As species. Significant variation in As concentrations in water and food can probably be related to differences in the geochemical characteristics of the environment (soil and groundwater), the specific As uptake capacity of crops, as well as food preparation and processing practices. The need to associate certain health effects with dietary intake of a particular contaminant, by establishing RP and MOE values (where applicable), is one of the most important tools for risk characterization in toxicological studies. As previously reported, some uncertainties/errors in risk assessment may arise from sampling and analytical techniques, in particular from speciation validation, from collection of consumption data over a few days, while long-term food consumption may not be appropriately represented, especially for rarely consumed foods, and from the failure to take into account differences in dietary habits of populations around the world. A harmonized approach using an intake estimation model that is as appropriate and realistic as possible is recommended.

## Figures and Tables

**Table 1:** Small methylated arsenic species [6].

Acronyms	Common Names
MMA(III)	monomethylarsonous acid
MMA(V)	monomethylarsonic acid
thio-MMA(V)	monomethylthioarsonic acid
dithio-MMA(V)	monomethyldithioarsonic acid
DMA(III)	dimethylarsinous acid
DMA(V)	dimethylarsinic acid
thio-DMA(V)	dimethylthioarsinic acid
dithio-DMA(V)	dimethyldithioarsinic acid
TMAO	trimethylarsine oxide
thio-TMA	thio-trimethylarsine
TETRA	Tetramethylarsonium

**Table 2:** Toxicity of inorganic arsenic in various systems/apparatus of human body [24].

Systems/Apparatus	Adverse Effects
Respiratory	Cough, bronchitis, trouble breathing, death due to lung disease
Gastrointestinal	Diarrheal events in children and topical lesions on the tongue and gums
Cardiovascular	Arrhythmia, hypertension, atherosclerosis, death due to ischemic heart disease and stroke
Endocrine	Diabetes mellitus
Nervous	Reduced velocity of peripheral nerve conduction, peripheral neuropathy, and impaired sensory function
Immune	Immune activity disorders with risk of infection in neonates and delayed hypersensitivity response in adults
Ocular	Eye disorders such as conjunctivitis, cataracts, and pterygium
Development	Fetal and neonatal deaths, congenital heart defects, growth and neurological development delays, increased susceptibility to infections
Skin	Hyperkeratosis, hyperpigmentation, or hypopigmentation (face, neck, and back)
Reproductive	Endometriosis, effects on the male reproductive system and sperm quality

**Table 3:** Maximum levels (mg/kg) of inorganic arsenic (iAs) in different food categories [35].

Food Categories	iAS [Sum of As(III) and As(V)]
Non-parboiled rice (polished or white)	0.15
Parboiled rice, husked rice, and rice flour	0.25
Rice products (breakfast flakes, waffles, wafers, crackers, cakes)	0.30
Rice products destined for infants and young children	0.10
Rice-based drinks	0.030
Powdered infant formulae and follow-on formulae	0.020
Liquid infant formulae and follow-on formulae	0.010
Baby food	0.020
Fruit juices and fruit nectars	0.020

## Data Availability

The original contributions presented in the study are included in the article. Further inquiries can be directed to the corresponding author.
